# Complete genome sequence of *Prevotella histicola* T05-04

**DOI:** 10.1128/mra.00144-25

**Published:** 2025-04-29

**Authors:** Kelyah Spurgeon, John R. Erb-Downward, Gary B. Huffnagle, Ariangela J. Kozik

**Affiliations:** 1 Department of Molecular, Cellular, and Developmental Biology, University of Michigan 118715, Ann Arbor, Michigan, USA; 2 Division of Pulmonary and Critical Care Medicine, Department of Internal Medicine, University of Michigan-Ann Arbor 1259, Ann Arbor, Michigan, USA; 3 Mary H. Weiser Food Allergy Center, University of Michigan 1259, Ann Arbor, Michigan, USA; 4 Department of Microbiology & Immunology, University of Michigan 242912, Ann Arbor, Michigan, USA; Rochester Institute of Technology, Rochester, New York, USA

**Keywords:** gram-negative bacteria, *Prevotella*, genomes

## Abstract

*Prevotella histicola* is a non-spore forming, obligatory anaerobic, gram-negative coccobacillus bacterium originally isolated from human oral squamous cell carcinoma tissue. Previously, the available genome for *P. histicola* T05-04 consisted of 109 contigs. Here, we report the complete (closed) genome sequence for *Prevotella histicola* T05-04 assembled using Nanopore sequencing.

## ANNOUNCEMENT

The bacterial *Prevotella* genus is comprised of many human-associated species that are highly abundant at different sites of the body. The ecological role of *Prevotella histicola* as a prominent member of the oral microbiome and its relationship with other members of the microbiome has yet to be fully understood. Genome sequencing of type-strain *P. histicola* T05-04 will aid in future investigations of the genomic features underlying the biological role(s) of this member of the human microbiome.

The *Prevotella* genus (phylum, Bacteroidota; class, Bacteroidia; order, Bacteroidales; family, Prevotellaceae) consists of gram-negative, obligately anaerobic, non-spore forming bacteria. We purchased lyophilized *P. histicola* strain T05-04 (DSM 19854), which was reconstituted using pre-reduced medium (Brain Heart Infusion Broth from Anaerobe Systems, cat# AS-872) under anaerobic conditions at 37°C.

Before sequencing, *P. histicola* T05-04 was cultured in Tryptone Yeast Glucose (TYG) medium (1, 2) and incubated at 37°C for 24 hours, streak plated onto Schaedler Kanamycin Vancomycin agar with 5% sheep’s blood for 24 hours all under anoxic conditions. A single colony was cultured in TYG media, incubated for 24 hours, and then verified by Gram stain, colony PCR, and 16S rRNA Sanger sequencing. DNA was isolated using the DNeasy Blood and Tissue Kit yielding 208 ng/µL using Qubit dsDNA HS Assay and a Qubit Fluorometer. 260/280 (1.89) and 260/230 (1.87) ratios were quantified using a Nanodrop One UV-visible spectrophotometer.

DNA library preparation was performed using Oxford Nanopore Technologies Ligation Sequencing gDNA–Native Barcoding Kit 24 V14 (SQK NBD114.24, ONT, UK) according to the manufacturer’s protocol and sequenced on a FLO-MIN114 Flow Cell (ONT, UK) for 1.5 hours. DNA fragments of all sizes were enriched with Short Fragment Buffer (SKQ 1481067) without shearing. Read lengths of N_50_ 1,670 and 720.55 k raw reads were generated using Dorado 7.4.14 with a minimum Q score of 12 after raw reads were generated.

The genome sequence was assembled using FLYE 2.9.5 (3) with default parameters. Genome polishing was performed using BWA (4) with default parameters and Racon (5) using mismatching and matching bases set to 3 and −3, respectively. A final round of polishing was performed using Medaka (6) with default parameters. Final assembly of *P. histicola* resulted in three circular contigs 2,176,075 bp, 812,616 bp, and 6,399 bp in length. *P. histicola* strain F0411 has been previously described to have two chromosomes of different sizes and divergent sequences (NCBI RefSeq assembly GCF_018128125.1), which we have identified is also true for *P. histicola* T05-04. In addition, the third circular contig (coverage >1,000×) likely encodes for a plasmid. Reorientation of each assembled contig was performed using dnaapler (7). Final visualization of the three contigs was performed via Bandage (8). fastANI (9) using default parameters demonstrated 97% identity to *Prevotella histicola*. Currently, *Prevotella histicola* genomes are assembled to the scaffold and contig level, with only one fully assembled genome available for a different strain (NCBI RefSeq assembly GCF_018128125.1). This underscores the significance of our work to fully assemble a closed genome for *P. histicola* T05-04.

The 3 Mbp genome for *P. histicola* T05-04 has a 41.5% GC content. Final assembly coverage averaged to 232× (contig 1 = 213×, contig 2 = 266×, contig 3 = 2,345×). Sequence annotation for the genome was performed using the NCBI Prokaryotic Genome Annotation Pipeline (10) predicting 2,110 protein-coding genes and 2,430 genes in total.

## Data Availability

All data are from this work is available under BioProject PRJNA1209967 and BioSample accession number SAMN46572951. Nanopore basecall metadata are submitted under SAMN46572951.
